# Biochar in the UK Print News Media: Issue Frames and Their Implications for Opening up Debate About Land-based Greenhouse Gas Removal

**DOI:** 10.1080/17524032.2024.2357318

**Published:** 2024-05-29

**Authors:** Carol Morris, Catherine Price, Brigitte Nerlich

**Affiliations:** a School of Geography, University of Nottingham, Nottingham, UK; b Institute for Science and Society, School of Sociology and Social Policy, University of Nottingham, Nottingham, UK

**Keywords:** Biochar, land-based greenhouse gas removal, opening up debate, print news media, issue framing

## Abstract

Biochar is a land-based greenhouse gas removal technology with potential to address the climate crisis. This article examines societal debate and discussion around biochar as represented in the UK print news media and reflects on its implications for the democratic governance of novel technologies. Using an “issue frame” analysis approach, the following frames are identified – Innovation, Economics, Security, Governance and Accountability, Risk, Justice, Substitution, Salvation and Tradition – with some more prominent than others. Economics and Innovation frames are particularly pronounced, together with the argument for market-based forms of governance, while Risk and Justice frames are weakly developed. The findings show that some frames and their associated actors dominate debate, while others are absent or side-lined. This might hinder opening up the debate to a wider group of stakeholders and publics and alternative framings, thus constraining effective governance of biochar.

## Introduction

In October 2018, the UN’s Intergovernmental Panel on Climate Change (IPCC) issued a stark warning about the scale and severity of the climate crisis and specified that urgent transitions are required in the land, infrastructure, energy, urban and industrial systems to ensure deep reductions in CO_2_ emissions. Alongside rapid decarbonization, the IPPC (2018), together with the UK’s Royal Society and Royal Academy of Engineering (2018), also recommends the deployment of greenhouse gas removal (GGR) technologies. GGR can take different forms including “engineered” approaches such as Direct Air Capture (DAC) and Carbon Capture and Storage (CCS), the latter associated with energy production and other industrial manufacturing processes. Also promising to remove carbon dioxide from the atmosphere are land-based GGR approaches. Somewhat distinct from their engineered counterparts they rely on relatively large areas of rural and other semi-natural land on which, for example, to grow trees or biofuel crops, restore peat, produce the feedstocks for and to deploy biochar, a type of charcoal that removes CO_2_ from the atmosphere and stores it in the soil. However, some land-based GGR methods have engineered dimensions: e.g. biochars produced through industrial pyrolysis methods and rocks for rock weathering GGR from quarrying. In the UK the potential of land-based GGR is being investigated through a £30 million “demonstrator” program funded by UK Research and Innovation (UKRI), a significant public investment. Biochar, rock weathering, peatland restoration, afforestation, and perennial biomass crops are the land-based GGR approaches within the demonstrator program, with biochar, as one relatively less familiar technology, providing the focus of analysis herein.

If biochar, together with other land-based GGR approaches, is to make a meaningful contribution to climate mitigation, it will not only have to demonstrate its technical efficacy. It will also need to enroll a diversity of constituencies, including from agriculture and other land-use sectors, together with communities located adjacent to and subject to the relative visibility and range of other potential impacts of the land being used by these GGR methods. However, it is unclear how these actors, together with those from civil society campaigning for climate change action, entrepreneurs and companies, scientists, policy makers, regulators, and wider publics, are responding to the risks and benefits of biochar. More broadly, the land-based GGR space is far from settled, not least because some of its techniques are relatively new to climate mitigation efforts. Some commentators and activists have already highlighted the risks of “mitigation deterrence,” or delays in reducing greenhouse gas emissions, arising from the deployment of any form of GGR technology (e.g. Carton et al., 2023; Markusson et al., 2022). “Mitigation deterrence” can become further complicated due to the blurring of the social and ethical boundaries between GGR technologies and mitigation (Cox et al., 2018). In part, this relates to how GGR technologies are framed in terms of benefits, risks, scale, and feasibility and how these framings are perceived by publics (Cox et al., 2018, 2020a; Waller et al., 2020). There is concern that when GGR technologies are framed as plausible and feasible this may reinforce and justify “business as usual,” whilst undermining the need for urgent reductions in carbon emissions (Colvin et al., 2020; McLaren et al., 2023).

With these questions and concerns in mind, the aim of this paper is to explore which actors are involved and how these actors are positioning themselves within UK biochar discussion and debate and what this might mean for the contribution of land-based GGR to climate change governance. The paper seeks to extend the somewhat limited social research into biochar which has explored farmer awareness and receptiveness (e.g. Clare et al., 2014; Latawiec et al., 2017), governance and carbon markets (e.g. Rittl et al., 2015; Ernsting et al., 2011; Shackley, 2014; Otte & Vik, 2017; Buck, 2019; Pourhashem et al., 2019) but has largely ignored broader societal understandings of and discussion around biochar.

Our investigation is guided by several questions. Which actors are more or less dominant and which actors are absent? What arguments are being made by these actors and what forms of expertise are used to make claims? How developed is discussion around biochar in terms of the breadth of actors, perspectives, claims, and expertise? Is there any consensus emerging or are there notable areas of disagreement and controversy? These questions matter because, as Science and Technology Studies has demonstrated, debates around new, potentially controversial scientific and technological developments need to be “opened up” to as wide a range of actors and expertise as possible (Stirling, 2008). This “democratising” of science and technology seeks to ensure that as many different positions and forms of expertise as possible are accounted for, and all potential risks, uncertainties and injustices are considered (Chilvers & Kearnes, 2016). Opening up enables a strong, accountable debate (Stirling, 2012), and with this comes the potential for reaching well-informed decisions about the most effective governance arrangements for climate mitigation.

The paper approaches its task through an analysis of the mainstream print news media as this provides a readily available resource to undertake a preliminary investigation of the public debate surrounding biochar in the UK. For our purposes, the media is understood primarily as providing a window *onto* biochar debate. However, it is recognized that the media is simultaneously a key actor *within* this debate which, through its editorial policies and other journalistic practices, can influence which other actors are heard, promoted or suppressed including those whose priorities and forms of reasoning support and contest land-based GGR technologies (Burgess, 1990; Burnard & Colvin, 2022; Colvin et al., 2020; Gamson & Modigliani, 1989). The dependence by journalists on “primary definers”^1^ (Hall et al., 1978; Harjuniemi, 2021) is one common practice that is particularly relevant to our interest in biochar actors and which of these is identified in reporting. In using the print news media as a means of accessing societal level discussion and debate around biochar, we acknowledge the relevance of, but differentiate our approach from, the body of allied, international scholarship that has explored media coverage of other, typically more heavily engineered, CCS technologies (Asayama & Ishii, 2013; Boyd & Paveglio, 2014; Buhr & Hansson, 2011; Feldpausch-Parker et al., 2013, 2015; Jiang et al., 2022; Luokkanen et al., 2014; Nerlich & Jaspal, 2013; Otto et al., 2022; Pietzner et al., 2014; Raimi et al., 2019; Wilson et al., 2009).

Our approach is inspired by Porter and Hulme’s (2013) study of geoengineering “issue frames” in the UK print news media which they propose as “a useful resource in guiding further research into perceptions of climate control^2^” (p.353). In the following sections, we provide background on biochar as one of a number of emerging land-based GGR approaches in the UK. We then discuss the issue framing methodology and its operationalization in analyzing debate around biochar – its actors, claims, arguments, and expertise – in four national broadsheet newspapers. Results are organized according to Porter and Hulme’s issue frames that were found to apply to biochar reporting – Innovation, Economics, Security, Governance and Accountability, Risk, Justice. Three additional issue frames unique to biochar – Substitution, Salvation, and Tradition – are identified that extend the original analytical framework while also providing insight into the distinctive range of biochar actors, arguments, and expertise. In concluding, we argue that the dominance of some issue frames with their associated actors, perspectives, and claims limits the opening up of biochar debate. This presents opportunities for social scientific research to investigate how a wider group of stakeholders and publics can contribute meaningfully to debate and decision-making around the use of biochar and other land-based GGR approaches in climate mitigation governance.

## Biochar: a relatively new land-based approach to greenhouse gas removal

A focus on biochar is justified because it is a relatively new and unfamiliar land-based GGR approach but one that has begun to attract attention from a widening group of actors including research funders, policymakers, scientists, and various industrial sectors (IPCC, 2022; Royal Society and Royal Academy of Engineering, 2018). The biochar demonstrator project, publicly funded through the UK’s GGR demonstrator program, began in May 2021, and includes field trials with arable farmers in England. It builds on at least two decades of international biochar research. The GGR demonstrator program is designed to inform GGR policy in the UK.

Biochar is a carbon-rich substance that is typically derived from plant materials including wood, forestry wastes, and agricultural crop residues. Other source materials can be animal and human wastes, municipal food waste, and invasive plants (Hansson et al., 2021; Otte & Vik, 2017; Pourhashem et al., 2019). It is produced through pyrolysis, a process that entails the thermal decomposition of biomass at high temperatures and under oxygen-deprived conditions (Otte & Vik, 2017; Saxe et al., 2019). The term “biochar” is a blend of “biomass” and “charcoal,” a lexical compound that was first used in the 1990s (OED, online), but this relatively recent date belies biochar’s long and complex history. Biochar is connected to terra preta, or Amazonian Dark Earths. These soils are small areas (2–20 hectares) in lowland Amazonia which contain large amounts of black carbon from the incomplete combustion of organic materials (Soentgen et al., 2017). Terra preta soils are not natural but were formed through human intervention between 2500 and 500 years ago (Price, 2021). Indigenous people burnt wood in hearths, and the carbonized remains were applied to the soils enabling the terra preta to form. Similar to terra preta are the African Dark Earths. These soils are still being formed as carbon is added to the soil in the form of “char” from hearths used for cooking and the production of palm oil and potash (Frausin et al., 2014).

Similar to the African context, biochar is already produced and used on a small scale in the UK by individual gardeners, allotment holders, and farmers using simple kilns. From a GGR perspective, proponents assert that biochar needs to be produced and deployed “at scale”. In the UK, the estimated potential of greenhouse gas removal for biochar is 6–41 MtCO_2_ per year, although globally, it is projected to be between 1.9 and 4.8 GtCO_2_ per year (Royal Society and Royal Academy of Engineering, 2018).

Biochar sequesters, for relatively long periods, a proportion of the carbon from its biomass source material that would otherwise be released to the atmosphere if that material was burned for energy production or left to decompose (Clare et al., 2014; Saxe et al., 2019). The exact length of time carbon is sequestered for is contested, with the International Biochar Initiative (2022) claiming 1440–14,500 years. Others claim the sequestration period is dependent upon feedstocks and the conversion process (Hansson et al., 2021) and there is also uncertainty about biochar’s ability to sequester carbon over long periods of time once applied to agricultural soils (British Society of Soil Science 2021). In addition to its potential to remove greenhouse gases from the atmosphere, biochar is also said to help improve soil health by increasing water and nutrient holding capacity with associated benefits to agricultural, horticultural, and also silvicultural crop yields (Otte & Vik, 2017; Pourhashem et al., 2019; Saxe et al., 2019). Biochar has also been investigated as an animal feed to establish if it has potential to improve livestock health, milk quality and to reduce ammonia emissions (Innovative Farmers, 2020). Although currently biochar’s use in food production contexts, both for GGR and crop yield benefits, is largely experimental, the National Farmers Union has identified biochar as one approach that may help the UK agricultural sector achieve net zero by 2040 (NFU, 2021).

In addition to its deployment in food and forestry production, biochar can also be applied within a variety of other land use contexts such as quarries, embankments, and golf courses where its benefits are understood to be carbon sequestration and land restoration (TerrAffix, 2022). Further, biochar is sold to domestic gardeners as a peat substitute (Carbon Gold, 2022). Biochar can be a part of alternative energy production systems such as bioenergy with biochar capture and storage and is also seen as a potential additive to road construction materials, or in cement and concrete production where the interest is to help “offset” the carbon emissions associated with these industries (Buck, 2019). Biochar can therefore be a waste management strategy, as part of energy production, as well as a material for keeping carbon in soils and a soil enhancer.

## Materials and methods

Using an issue frame approach based on Porter and Hulme’s study of geoengineering (2013), we explore the emerging societal level discussion and debate around biochar through its reporting in the UK print news media. Despite the rise of social media and other news sources (Harcup & O’Neill, 2017), the “traditional” print news media has continuing significance in media discourse, justifying its use as a vehicle for accessing biochar debate, its framings, and associated actors. Frame analysis of one variety or another has a long and well-established tradition within media and communication studies (Entman, 1993; Gamson & Modigliani, 1989; Goffman, 1974; Snow & Benford, 1988). It has also been used in scientific scholarship including rural-environmental studies focused on land use matters (e.g. Kirwan & Maye, 2013; Mooney & Hunt, 2009; Morris et al., 2016; Rivera-Ferre, 2012).

To frame means to emphasize and make resonant particular facets of an issue over others (Entman, 1993). More specifically, a frame, following the work of Snow and Benford (1988), provides a diagnosis of a problem, specifies an impetus or motivation for acting on that problem and offers a prognosis including recommended solutions to the problem. A framing process is partial and lacks neutrality, as actors with particular interests and agendas mobilize some framings rather than others in order to influence public opinion and policy agendas particularly when topics are unfamiliar or of concern as can be the case with new technologies such as biochar (Boyd & Paveglio, 2014).

Following De Vreese (2002), Porter and Hulme (2013) distinguish between “issue specific” frames – which are only relevant to particular topics or events – and “generic” frames – which transcend thematic limitations over time and space. They make the case for focusing on the former when trying to make sense of geoengineering media discourse because issue frames “generally offer greater insight into the issue-specific discourse” (p. 344). Although some forms of frame analysis suggest that the number of frames is likely to be limited (e.g. Benford and Snow’s 2000), others place no limit on the number of frames (e.g. Cappella & Jamieson, 1997), with Porter and Hulme identifying seven issue frames in the reporting of geoengineering in the British news media: Innovation, Economics, Security, Governance and Accountability, Risk, Justice, Morality.

To explore these issue frames, their associated actors, arguments, and expertise, within biochar reporting we searched the news database Lexis Nexis on 4 March 2022. In All English Language News, over 10,000 articles mentioning biochar have been published overall since 2007. Of these, 408 articles were published in British newspapers, with the first article being published in 2008. We selected the newspapers listed as the top four publishing on biochar, all broadsheets but of different political orientations, with *The Guardian* on the left, *The Daily Telegraph* on the right, with *The Independent* leaning toward the left and *The Financial Times* being seen as center-right. Together, these newspapers had published a total of 100 articles, a number suitable for in-depth qualitative analysis: *The Guardian* (*N* = 54 articles; first article 2008), *The Daily Telegraph* (*N* = 19 articles; first article 2009), *The Independent* (*N* = 14 articles; first article 2009) and *The Financial Times* (*N* = 13 articles; first article 2008). Full details of cited articles are provided as supplemental material. We acknowledge that tabloid newspapers have a larger readership, but as they had published so few articles on biochar their inclusion was judged to be not worthwhile.

Porter and Hulme inductively developed their issue frames based on grounded theory which allowed the frames to emerge without reference to a pre-existing theory. As we worked with their frames, to test their relevance to biochar as a climate technology, our approach was deductive, searching for evidence within our corpus of each issue frame together with its constituent actors. For example, evidence for the presence of a “Governance and Accountability” issue frame was identified in discussion of key governance domains notably the state, market, and civil society, interventions such as specific policies or legislation together with the actors cited as shaping governing practices. Evidence for an Economics frame was found in references to the financial, business, and macro-economic dimensions of biochar production and deployment and relevant actors, such as individual entrepreneurs and economists. We found only limited evidence for some of the issue frames identified by Porter and Hulme, notably Security, Justice, and Morality, and other aspects of the material could not be made sense of through the pre-existing issue frames. For these sections of the data an inductive approach was employed to enable the identification of additional issue frames guided by Cappella and Jamieson’s (1997) frame criteria (as used by Porter and Hulme): identifiable conceptual and linguistic features; commonly observed^3^; easily distinguished from other frames; recognisable by others. With respect to each of these criteria the three researchers coded a sample of the corpus to ensure a consistent approach to the identification of both the pre-existing, but also three additional issue frames – Tradition, Salvation, and Substitution – distinctive to the biochar debate and its reporting. In the section that follows we first provide a brief overview of the variation in levels of biochar reporting before presenting each of the issue frames in turn.

## Results

**Figure 1.**
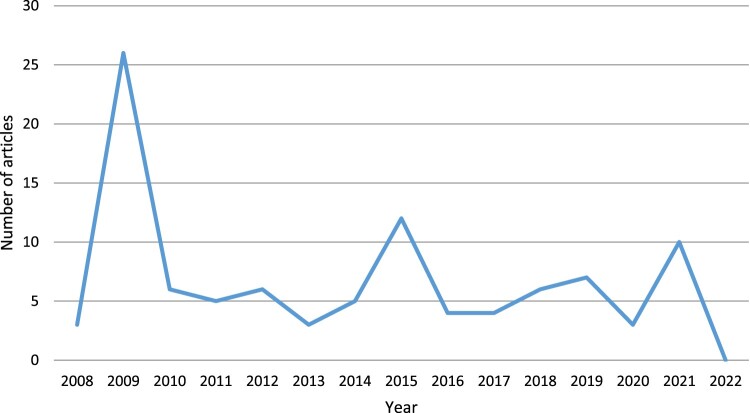
Number of UK national broadsheet biochar news articles by year.

The number of biochar articles published in the UK national broadsheets has varied across the years (Figure 1). The first article appeared in 2008 with no articles appearing in 2022 (until 4 March 2022 when the dataset was extracted). The peak of reporting occurred in 2009 when 26 articles were published. Two smaller peaks occurred in 2015 (12 articles) and 2021 (10 articles).

In the peak of 2009, most articles relate to a piece authored by the environmentalist George Monbiot in March 2009. Monbiot’s critique of biochar resulted in a flurry of reports either supporting his view or defending biochar. The increase in reporting in 2015 or 2021 may be coincident with the Paris and Glasgow climate summits in each of these years which inevitably prompted discussion of solutions to climate change.

### Analysis of issue frames – their actors, arguments and expertise – in the news articles

The issue frames are presented in descending order of prominence, drawing out key associated actors, arguments, and expertise. Table 1 provides an overview of the characteristics of each frame.

**Table 1.** Overview of issue frame characteristics, actors and expertise.

Issue frame	Frame characteristics	Key actors	Actor expertise (explicitly stated and implied)
Innovation	Biochar as a simple, low cost and accessible climate innovation attracting research interest and funding.	Scientists, research institutes, and biochar entrepreneurs engaged in biochar research; gardening experts and horticultural organizations “trialling” biochar.	Scientific knowledges dominate; limited acknowledgement of non-certified expertise e.g. held by global south farmers; practical / experiential knowledge of gardening; business knowledges.
Economics	Biochar as a business and economic opportunity and cheap solution to climate change. “Scaling up” is a challenge but also an investment opportunity.	Scientists; gardening / horticultural practitioners; environmental and gardening journalists; entrepreneurs; representatives of the finance industry including venture capitalists; environmental NGOs.	Scientific knowledges; practical / experiential knowledge of gardening; business, financial and economics knowledges both orthodox and alternative (e.g. ecological economics) to a much lesser degree.
Security	Biochar as contributor to or detractor from societal security with respect to climate change.	Scientists; environmental NGOs.	Environmental science knowledges
Governance and Accountability	Climate innovations such as biochar will require new governance arrangements, with carbon markets key to “scaling up”.	Venture capitalists; climate tech (including biochar) entrepreneurs; scientists, particularly climate scientists; environmental NGOs; gardening journalists.	Orthodox economic knowledges; scientific knowledges.
Substitution	Biochar as a beneficial alternative to peat in the context of domestic gardening.	Climate tech (including biochar) entrepreneurs; gardening journalists; plant scientists.	Scientific knowledges; practical gardening knowledge; business / economic knowledges.
Risk (and uncertainty)	Biochar presents environmental risks, alongside its potential benefits. Uncertainties are ongoing and used to justify further biochar research.	Environmentalists; environmental scientists; plant scientists; gardening experts; environmental journalists.	Environmental science knowledges.
Tradition	Biochar as a traditional material inspired by the, ancient dark earths or Terra preta soils of South America and Africa	Social scientists; biochar entrepreneurs; gardening experts; environmental journalists.	Social science knowledges; practical knowledge of gardening; Indigenous knowledges
Salvation	Biochar as savior of soils, trees and planet.	Scientists; gardening experts; environmental journalists.	Scientific knowledges; practical knowledge of gardening.
Justice	Biochar as a potential cause of distributive injustice.	Scientists and research organizations; environmentalists and environmental NGOs.	Environmental science knowledges; Indigenous knowledges.

#### Innovation

An Innovation framing was the most pronounced, with half of the articles explicitly describing biochar as a climate innovation (e.g. Crooks 2009; Shemkus 2015). An Innovation framing is particularly apparent in the attention given to biochar research as a key dimension of innovation processes. Many articles discuss recently published research reports that focus on or mention biochar (e.g. Dudman 2010) and/or that feature individual scientists, research institutes, and entrepreneurs engaged in biochar research.

The contribution of gardening experts and horticultural organizations in biochar “trials” designed to demonstrate the benefits of biochar to gardeners is a distinctive feature of this issue frame and involves the mobilization of both scientific and practical/experiential gardening knowledges while also adding further insight into the many sites of biochar experimentation (e.g. Preston 2019). Faith in science and the role of certified knowledge is clearly prominent in the Innovation frame, a feature also observed by Porter and Hulme (2013), together with practical / experiential knowledge in a gardening context. Some recognition is given to biochar knowledge co-produced with non-certified experts particularly farmers in the global south (e.g. Leendertz 2013; Guardian 2009b).

Other scientific and technological developments are addressed within this Innovation frame when, for example, biochar is folded into wider discussion of renewable energy production (e.g. Cookson 2009). But unlike the “techno-pessimism” evident in media debates surrounding “high tech” geoengineering technologies (Porter & Hulme, 2013) biochar is more likely to be represented as a preferable innovation for tackling climate change because of its apparent simplicity, low cost, and accessibility. As Craig Sams, a British entrepreneur whose company Carbon Gold has commercialized biochar for use by UK gardeners explains:

It's not big, shiny steel stuff - the [Carbon Gold biochar] units cost a few thousand pounds or less … You don't need a degree in engineering to keep it from exploding or catching fire (Harries 2009).

Although Sams goes on to argue that extensive research investment is not required, the Innovation frame emphasizes the ongoing importance of research and experimentation in biochar’s development, with scientists dominating discussion together with gardening and horticultural experts and some entrepreneurs.

#### Economics

Evident in at least half of the articles, and consistent in reporting across the period, is an Economics framing. Those featuring scientists address the costs of biochar, with some articles claiming that the technology is a relatively cheap solution to climate change (e.g. Goodall 2008; Connor 2015), compared with other forms of carbon capture and energy generation. Biochar is depicted as a relatively more expensive option for gardeners seeking peat alternatives (e.g. Nex 2021; Walker 2012) in articles authored by gardening journalists or featuring gardening experts. There is wide acknowledgement within this framing that there are a variety of business opportunities in biochar manufacture and deployment. The potential of the biochar industry to support national and international economies is asserted by businesses (e.g. Asprem 2009) whilst shifts toward “low carbon economies” are endorsed by environmental journalists (e.g. Harvey 2009). Biochar is promoted by scientists as a means by which landowners can generate income from the manufacture and sale of biochar (e.g. Read 2009) while entrepreneurs advocate the selling of carbon credits by landowners (e.g. Sams 2018). Gardening and environmental journalists endorse biochar’s ability to both reduce fertilizer costs and increase crop yields (e.g. Vernon 2011; Harvey 2019).

The excitement surrounding the business opportunities of biochar is nuanced by recognition that the biochar industry is in its early stages of development and that if it is to realize its promises then biochar production needs to significantly “scale up” (Flannery 2015a). As researcher Donna Udall explains:

Only when farmers, food producers and other industries start putting their biomass waste through a pyrolysis process as standard would there be enough biochar to roll it out on a wide scale (Murray 2019).

The development of carbon markets, in which biochar is made eligible for carbon credits, is identified as a key mechanism to enable the industry’s scaling up (see Governance and Accountability frame).

The challenge of attracting sufficient investment in the embryonic biochar industry, and in other so-called “climate tech” industries, is an allied dimension of the scaling up narrative and a further feature of articles employing an Economics framing. However, this is typically presented as an opportunity for investors including venture capitalists and some scientists making the case for more biochar investment (Flannery 2015b; Thornhill 2020). An additional challenge identified for the biochar industry is getting “conservative” farmers to adopt this new product (Shemkus 2015). However, this does not feature prominently in the reporting which otherwise presents biochar as more immediately relevant to (domestic) horticulture i.e. gardening rather than commercial agriculture and links this frame to the Substitution frame described below.

Undeveloped within the Economics frame is discussion of biochar, along with other GGR approaches, as a distraction from the need to make more fundamental changes to political economies to address climate change. This is an argument that is much less pronounced than in Porter and Hulme’s (2013) analysis of geoengineering in the media, appearing in only a handful of articles and only in the left wing and centrist press. It features, for example, in the claim made by environmental NGOs that biochar-based carbon capture represents a problematic “Business as Usual” economics, an argument also made by environmental economists and some scientists (Ahmed 2014; Nogrady 2017).

Given the marketization of UK society in recent decades (Harvey, 2005), it is perhaps unsurprising that the Economics framing of biochar is not only pronounced within the debate but associated with a diversity of actors (some of whom, perhaps unwittingly, emphasize the economic dimensions and benefits of biochar) with a breadth of expertise from different forms of science, economics, finance, and business. Less pronounced are “alternative” perspectives from different schools of environmentalism and non-orthodox economics (as also observed by Harjuniemi 2021).

#### Security

The Security frame conceives anthropogenic climate change as a threat to societies, and mainly appears around 2009 when a breakthrough to large-scale deployment in agriculture seemed imminent (Bezerra et al., 2019; Schmidt & Shackley, 2016).

Biochar either contributes to or detracts from societal security by offering a potential solution to or worsening climate change. As a potential means of securing futures scientists and scientific journals feature as prominent actors within this frame by presenting an optimistic view of biochar. Chris Turney, a professor of geography at the University of Exeter, said that “biochar was the closest thing scientists had to a silver-bullet solution to climate change” (Guardian 2009a). While stressing that biochar was only one part of the solution, Marc Redmile-Gordon, senior scientist at the UK’s Royal Horticultural Society argues: “If we stop burning fossil fuels tomorrow, we'll still have a lot of carbon dioxide removal to be doing, and this is one of the most effective ways we can achieve that” (Murray 2019). Although a tempered perspective, the optimistic framing of biochar as a contribution to societal security was still evident.

However, biochar could potentially be a threat to addressing climate change and was framed as a threat to security most prominently by environmental NGOs. Nick Rau of Friends of the Earth, views biochar as a “fake solution” (Harries 2009). Similarly, Deepak Rughani from Biofuelwatch warns that although biochar has been “Widely touted as the most promising geo-engineering “solution”, [it] … actually threatens to commit us to even more dangerous climate change” (Rughani 2009).

#### Governance and accountability

Less than one fifth of articles were interpreted as within a Governance and Accountability frame addressing the institutions and political mechanisms through which biochar would and should be governed. A larger number examined the broader challenges of governing climate change and the governance of other activities relevant to biochar e.g. agriculture. These reports often appear to be linked to international governance events such as climate conventions, associated national level policy measures and whether these interventions are adequate for the task of addressing climate change and / or may entail knee jerk policy responses supporting unproven GGR technologies including but not limited to biochar (Nogrady 2017). Nevertheless, these government led efforts are typically presented as key to providing an enabling regulatory environment in which GGR developments can take place whilst working in partnership with the private sector in the effective governance of climate change (e.g. Thornhill 2021). These include scientists (see Nogrady, 2017), and a variety of business actors, including venture capitalists.

Within the biochar-specific reporting, the Financial Times is unique in referring to the absence of biochar regulation in an article with a gardening focus and written by a gardening journalist (Bergloff 2011). The proper control of biochar’s production – “what it's made from and how” – is argued to be necessary to realize biochar’s “potentially great contribution in mitigating soil depletion and CO2 emissions”. Otherwise, discussion about biochar governance focuses on market-based forms of regulation and in particular the perceived need for biochar to qualify for carbon credits so that it can be included in carbon trading schemes. These governance mechanisms are cast as vital to the “scaling up” of the biochar industry (Guardian 2009a). A prominent figure in making the case for biochar’s qualification for carbon credits is entrepreneur Craig Sams, with the scientific organization the International Biochar Institute and some science writers also identified as proponents. Sams argues:

Once producers are rewarded for sequestering carbon [through carbon pricing] they will change their behaviour. That's the carrot. A stick might be a requirement to compensate society for the future harm they are causing by contributing … 25 per cent of annual greenhouse gas emissions (Sams 2018).

The case in favor of governing biochar through market mechanisms dominates the Governance and Accountability frame which, as with the Economics frame, may at least be partially explained by a prevailing neoliberal ideology. Only a handful of earlier articles present a more critical perspective with one claiming that soil-based carbon credits have found “little favor” amongst climate scientists (Harvey 2009). Other featured actors provide more forthright critiques, with Deepak Rughani of Biofuelwatch (2009) warning that the case for biochar carbon credits to be included in the Clean Development Mechanism by biochar industry lobbyists is “fairytale spin”. After these articles, published in the early stages of the analysis period, the negative press surrounding biochar carbon credits disappears even though reporting on the topic continued. This coincides with the period when biochar emerged as a potential GGR technology. Biochar can be marketed as an approach of creating revenue if carbon can be traded on biochar carbon markets (Bezerra et al., 2019).

The Governance and Accountability frame is dominated by climate technology entrepreneurs and venture capitalists with support from certain scientists. This aligns with the Economic and Innovation frames. Appearing less frequently are climate scientists and environmental NGOs who, when they do, are critical of market-based mechanisms to biochar’s governance.

#### Substitution

The Substitution frame is one of three issue frames distinctive to biochar reporting and presents biochar as a beneficial alternative to peat for gardeners. When peat is extracted, carbon is released into the atmosphere, thus contributing to climate change. The UK government is currently phasing out peat-based composts, and by 2024, all sales of peat to amateur gardeners in England will be banned (UK Government, 2022), requiring gardeners to seek alternatives. The Substitution frame was evident in 13 articles, appearing predominately in 2011 and 2012. Terms used to describe biochar as a peat substitute include “wonder material” (Bergloff 2011) and “miracle product” (Thompson 2016).

Articles featuring a Substitution framing present biochar, its GGR and soil health properties, as something that is more immediately familiar to readers i.e. through gardening. For example, the gardening author, Lia Leendertz writes (2013), that when peat is extracted from peat bogs, carbon sequestered thousands of years ago is released into the atmosphere. As biochar can sequester carbon, those gardeners who use it are helping address the environmental damage caused by those who use peat. This claim was further endorsed by Craig Sams, through the promotion of his Carbon Gold products in three separate news articles with the final one claiming that biochar is “as close to peat as you can get. It's sustainable, doesn't require the destruction of peat bogs and actually lasts far longer in the soil” (Diacono 2011). In the Substitution frame biochar has environmental credentials whilst peat is destroying the planet.

Although the Substitution framing almost exclusively presents biochar as a beneficial substitute for peat, it was also reported as a costlier alternative. Ken Thompson, a plant ecologist and gardening columnist, explains that whilst biochar can improve the moisture-holding capacity of soil, and increase the pH of some acidic soils, it is expensive (Thompson 2016, also Walker 2012).

#### Risk

A risk frame is only used in approximately 10% of articles, far less than in Porter and Hulme’s analysis of geoengineering in the media but is a consistent feature throughout the period of analysis. Further, risk discourse is not presented as a function of probability and impact, and as “matter-of-fact,” but instead is ethically charged (linking this frame to the Justice frame). The difference in the discourse can be attributed in part to the actors involved. In the geoengineering case the Royal Society is the most prominent actor when risk is discussed but with biochar it is environmentalists who typically mobilize scientific data (Eden et al., 2006).

The overarching theme which appears in the Risk frame is the potential of biochar to increase carbon emissions, and hence worsen anthropogenic climate change, because trees (which are already storing carbon) are burnt to produce a material that stores carbon. As one article claims, there is “nothing green about logging and burning native forests” (Cox 2021). Mike Childs, climate campaigner with Friends of the Earth, compares biochar to biofuel and argues that at small scale it contributes to climate change mitigation but at large scale not (Guardian 2009c).

Interacting with the Risk framing, and slightly more prominent (appearing in 15 articles), is uncertainty, a feature of geoengineering’s media coverage where the possibilities of unforeseen and unintended consequences make it difficult to calculate risk (Porter & Hulme, 2013). With biochar, attention is focused – mostly by individual scientists and science organizations – on the uncertainties surrounding whether and to what extent biochar will have its desired impacts in terms of GGR and / or as a soil amendment. For example, scientist Johannes Lehmann (in Harvey 2009) described the uncertainty surrounding the amount of carbon that biochar could remove from the atmosphere. Meanwhile, in an article about green gardening an ecologist and gardening columnist raises doubts about biochar’s ability to enhance soil fertility: “The bottom line is there's not much evidence that it does anything. … If you start with a pile of wood with no nutrients, turning it into biochar won't add any extra” (Nex 2021).

Actors from the scientific community dominate uncertainty discourse which gives the impression that the scientific community are not yet agreed about biochar. This persists throughout the period of analysis with earlier and more recent articles highlighting that research has not yet produced a sufficient body of evidence to enable policy decisions about biochar’s deployment in agriculture and horticulture. Science writer Chris Goodall is quoted as saying, “No one argues that biochar's effects are well understood. Scientific investigation is only just beginning” (Monbiot 2009a). In a later article, the Royal Horticultural Society advises gardeners to adopt a cautious approach to “new” soil additives as there is, as yet, not enough evidence for or against (Telegraph 2016).

Although biochar research is reported by environmental journalists as having made progress and produced promising findings (e.g. Harvey 2019) considerable uncertainties are claimed to remain, justifying further research.

#### Tradition

A “Tradition” frame is distinctive to the biochar reporting and a framing through which biochar’s production and use is legitimized. This framing depicts biochar as a material inspired by the traditional, ancient dark earths or Terra preta soils of South America and Africa. However, it is a frame that only appears in seven articles and is relatively short-lived with first mention in the Financial Times in January 2009 and last reported in the Telegraph in April 2013. Once biochar emerges as a potential GGR technology in the late 2000s, the connection to terra preta soils dissipates. Biochar is instead put forward as a modern, techno-scientific, and universal technology which is far removed from the local Indigenous knowledge and practices associated with terra preta soils (Bezerra et al., 2019).

The environmental journalist, Fiona Harvey, explains in 2009 that the Terra preta soils in Brazil’s Amazon basin were formed between 2500 and 6000 years ago. She goes on to describe how the soils are not natural but were created over this extended period by Indigenous peoples. Harvey writes:

These cultures survived and supported complex agriculture, despite poor soil, by making their own earth. They used dung, fish, animal bones and plant waste – the usual suspects. But the key ingredient in terra preta, and what gives it its dark colour, is charcoal.

In the same news article, Simon Shackley, a social scientist from the University of Edinburgh, also acknowledges that the soils were created as a result of slash and burn agriculture and the addition of organic waste. This variety of added materials is claimed to enhance the fertility of the Terra pretas, soils that have not been successfully recreated. The Terra preta soil is reported as a key inspiration behind the establishment of Craig Sams’ Carbon Gold biochar company.

The Tradition frame is also mobilized when ancient techniques are referenced in the promotion of biochar products. In an article focused on enhancing soil health in gardening written by Jean Vernon (2010), a gardening correspondent for The Telegraph, attention is drawn to two biochar products. Firstly: “Takesumi Carbonised Bamboo Biochar […] was inspired by the oriental use of charcoal as a water purifier and soil additive”. Secondly: “Carbon Gold GroChar Soil Improver […] was inspired by Amazonian Indians who use charred plant matter to enrich infertile soils. This terra preta – or dark earth – remains fertile thousands of years later”.

The Tradition frame is dominated by actors who have the scientific and / or practical expertise to discuss soil and soil properties.

#### Salvation

A further frame distinctive to the biochar reporting is that of “Salvation”. It is used throughout the duration of the study, with no particular time period dominating. However, it only appears in four articles. Here, biochar is a material that can potentially save degraded soils, trees, and even the planet. This framing is found in headlines such as: “This gift of nature is the best way to save us from climate catastrophe: Biochar schemes would remove carbon from the atmosphere and increase food supply” (2009) in an article authored by scientist Peter Read; and “A slow-burn success: Biochar could help you get more from your plants and save the planet at the same time” written by gardening author, Lia Leendertz (2013). Framing biochar as salvation is also carried over into the body of the news articles, with Harvey (2009) writing that “the carbon-capturing charcoal […] might, just might, save the world”. This framing is dominated by those whose expertise is more environmentally focused.

#### Justice

The normative dimensions of biochar reporting, shaped by ethical judgements about winners and losers, are limited but more evident than a Morality framing. The latter, according to Porter and Hulme (2013), is concerned with the relative “rightness” and “wrongness” of deploying, or not, a particular GGR technology and features religious connotations, references and language. Since we could not find sufficient evidence of this type of reporting we only include discussion of a Justice framing of biochar while acknowledging that this framing includes reference to “rightness” and “wrongness” and thus closely aligns with a Morality framing.

The core objection within the Justice framing is that growing biomass in the form of forests, either existing or planted on an industrial scale, to produce biochar is in tension with the use of that land for food production and / or by Indigenous, subsistence communities. In this way, biochar production is framed as similarly problematic to biofuel production in that it competes for agricultural land and the loss of that land to biomass is likely to increase food prices with detrimental consequences for the poorest members of society (Ahmed 2014). Discussion of these potential distributive injustices of biochar appeared in a handful of articles and only in the early part of the period of analysis (Asprem 2009; Harries 2009). Actors include Mads Asprem (2009), the managing director of Green Resources, Dr Rachel Smolker, Biofuelwatch, Danielle Pafford, Centre for Alternative Technology (Ahmed, 2014), and Nick Rau, Friends of the Earth (Harries, 2009). This time period again corresponds with the emergence of biochar as a potential GGR technology, along with the disconnection of biochar from terra preta soils.

The strongest opposition regarding the distributive injustices of biochar production came from the environmentalist George Monbiot in response to a news article authored by Peter Read of Massey University, New Zealand. The first part of Monbiot’s response focuses on those living on so-called “uninhabited” and “degraded land” seen as suitable for plantations, namely “subsistence farmers, pastoralists or hunters and gatherers” (Monbiot 2009b). The second part of the response refers to “unused potential arable land” that Read would probably cover with “sitka spruce plantations that blight the lives of everyone who loves the countryside here” (Monbiot 2009b).

In a retort, Read claims that the land he identified was not inhabited by the groups Monbiot mentions but “logged over and abandoned” and he asks: “Given the chance, impoverished people often opt for a waged income. Does Monbiot wish to keep them impoverished for ever?” (Read 2009). After this exchange, discussion of biochar’s potential (in)justices almost disappears from the media coverage revealing that these disbenefits have become less pronounced over time as the Economics and Innovation frames have come to dominate discussion.

## Discussion and conclusions

This paper has explored societal level discussion and debate around biochar in the UK, a relatively new and unfamiliar land-based GGR technology, as this is publicly expressed and mediated through the mainstream print news media since first reports in 2008. This type of study is situated within a broader trend in knowledge production to not only examine the technical dimensions of a new technology – whether it “works” in practice – but also whether it “works” for society in terms of acceptability and responsibility. Methodologically we have been guided by Porter and Hulme’s (2013) analysis of geoengineering issue frames in the UK print news media, an approach that enables systematic investigation of the multiple ways in which biochar is being framed, by whom and how, the relative prominence of these different issue framings and what they reveal about the nature and degree of consensus and contestation around biochar as a land-based GGR approach. In this final section, we highlight key findings, with a focus on our central question as to whether biochar debates (as represented in the press) are opening up a space for societal debate. We end by making recommendations for future research.

Some issue frames, their mobilizing actors, arguments, and expertise, clearly dominate over others notably the Economics and Innovation frames which together emphasize the innovativeness, economic and financial benefits of biochar, foreground entrepreneurs and research institutes and scientists, and make a positive case for biochar as a land-based GGR approach. The techno-optimism evident within biochar discussion are in line with many other studies of CCS technologies in the media including those from North America (Wilson et al., 2009; Feldpausch-Parker et al. 2011, 2013, 2015; Boyd & Paveglio, 2014), Norway (Buhr & Hansson, 2011), China (Jiang et al., 2022), Japan (Asayama & Ishii, 2013) and in geoengineering narratives in Australian online media (Burnard & Colvin, 2022). The most prominent biochar issue frames foreground market-based approaches to governing the embryonic biochar industry to facilitate its scaling-up. The case for other governance approaches located, for example, within the local and national state and civil society are largely absent and so too, by extension, are actors linked to these other governance domains. These emphases within biochar debate are linked to a particular group of elite actors, thus limiting the inclusion of other actors and voices and restricting the opening up debate about biochar.

Another key finding is the identification of additional issue frames that did not feature in Porter and Hulme’s (2013) analysis. These issue frames of Tradition, Substitution, and Salvation are linked to specific groups of actors and forms of expertise and are clearly associated with the land-based qualities of biochar as derived from biological materials and deployed to soil, and a soil like substance. Further, they all contribute to biochar’s legitimation rather than its problematization as a potential GGR approach and emphasize biochar’s use in domestic horticulture rather than in other land use contexts including agriculture. Biochar is presented as a material which is caring for the planet and can be used easily by gardeners. This simple, low-tech solution potentially disrupts the vision of more highly engineered GGR approaches. Given this framing, it is not entirely clear why biochar has to date captured the imagination of gardening rather than agricultural, forestry and other land-use actors but lack of awareness and understanding, “conservative attitudes” (Shemkus 2015) and lack of meaningful incentives (from any governance domain) may offer some explanations while also signaling the need for further investigation. And again, this one-sided domination of framings excludes other voices and actors.

Although present in the reporting, the Risk and Justice issue frames are very weakly developed, while a Morality framing, as identified by Porter and Hulme (2013), is not present at all. These “counter” frames are linked to a somewhat different group of actors, notably environmental NGOs, environmental economists, and scientists, who offer alternative perspectives on biochar that question its contribution to climate mitigation, including on grounds of environmental concerns and land-use conflicts (biochar feedstocks vs food production and /or afforestation for carbon sequestration). The presence of these actors and their claims-making about the risks, uncertainties, and injustices of biochar introduce dissenting voices that oppose the dominant issue framings, yet these voices are not very prominent which limits their potential contribution to an opening up of discussion and debate.

The analysis shows that certain frames, such as Economics and Risk, consistently feature through the period of analysis. However, for frames such as Security or Tradition, these only feature for a short period. These time periods appear to correspond with the moment in which biochar emerged as a potential GGR technology. Given the limited number of articles appearing in frames such as Salvation or Justice, it is difficult to provide a more extensive analysis. Further research could therefore focus on a more in-depth analysis of temporal variation within the frames.

Actors that one might expect to find within biochar discussion and debate are notable instead by their absence or their very limited presence. These are agricultural actors and other rural / land-use actors (that may be reasonably expected to appear more prominently in the Economics and Innovation framings), a wider group of environmental NGOs, and wider publics / citizens. The latter two groups of underrepresented actors have also been reported as less visible or absent in some other studies of climate technologies in the media (Asayama & Ishii, 2013; Buhr & Hansson, 2011; Jiang et al., 2022; Otto et al., 2022) but might be expected to be aligned with any or all of the Governance and Accountability, Risk, Justice and Morality framings, highlighting risks, uncertainties and injustices surrounding biochar and the acceptability of biochar production and use. Whilst there is mention of biochar feedstocks in terms of land use and the parallels with biofuels, there is no reporting around possible biomass feedstocks, their actual and potential availability and place(s) of production, or how these feedstocks should be transported. For commercial scale production, pyrolysis plants will be required to produce biochar but public acceptance and planning permission are not discussed. The absence of these issues in the media coverage provides an insight into how the biochar debate is not (yet) being fully opened up.

The analysis herein offers only a preliminary insight into biochar debate, its constituent actors, arguments, and expertise, since it has been accessed through coverage in the print news media rather than through primary, direct investigation of identified actors, approached, for example, through a systematic stakeholder mapping exercise (e.g. Raum, 2018). This is a limitation of the study. Nevertheless, the findings are revealing, as the balance of evidence points toward a somewhat limited or partial opening up of discussion and debate around this emergent climate technology. The current prominence of some actors, perspectives and expertise, at the possible expense of others, reduces confidence in decision-making around biochar’s contribution to climate mitigation governance. This signals a need for further research.

Firstly, are the actors who appear in the print news media reflective of what is happening in the biochar landscape in reality? Alternatively, are the actors appearing in the print news media doing so because of journalistic bias toward the biochar debate in which a reliance on primary definers (Hall et al., 1978) may be playing a part? These questions could be addressed through stakeholder mapping on the one hand and, on the other through interviews with journalists to establish why they have reported certain actors, and at particular moments in time, in print news media in relation to biochar.

Secondly, research could include a role for stakeholder and wider public engagement activities to help further open up the debate. Such activities need to be designed in ways that avoid a closing down of debate, as observed in public engagement with other GGR approaches including CCS (Mabon et al., 2015). Mabon et al. (2015) suggest the aim of public engagement work on actual CCS projects and some large academic research projects is to facilitate deployment, with a lack of meaningful debate about whether it is acceptable to stakeholders. Only small, narrowly framed details are left for stakeholders to discuss. To avoid this, stakeholders including from agriculture and other land-use sectors and wider publics need to be provided with the opportunity to offer alternative framings of biochar (Bellamy et al., 2013; Bellamy & Lezaun, 2017; Cox et al., 2020b). A diversity of perspectives can aid decision making, assist in the co-creation of knowledge and responsibly develop (Cox et al., 2020b) biochar as a land-based GGR approach.

## Supplementary Material

Supplementary Material

## Notes

1 Primary definers are journalists” “go to” individuals and institutions – typically accredited political representatives and knowledge producers – and as such are given privileged access to the media. The dependence by journalists on primary definers leads to a tendency within the media to disseminate and reproduce elite ideas while overlooking or actively downplaying alternative perspectives.

2 Biochar is not widely conceptualized as a technology of “climate control” but it has been discussed under the geoengineering banner (Royal Society, 2009) alongside solar radiation management (SRM) which is a controversial technique aiming to modify the Earth's albedo (Lomax et al., 2015; Buck, 2018). Proponents of GGR approaches argue that these could play a valuable role in climate mitigation efforts, but they have little in common with SRM (Lomax et al., 2015; Meadowcroft, 2013). GGR technologies now tend to be decoupled from geoengineering discussions (Lomax et al., 2015).

3 In Porter and Hulme's application, “commonly observed” meant that an issue frame was identified in more than 10% of articles. In this study, two of the additional issue frames – Tradition and Salvation – did not meet this criterion. However, we include them because they otherwise met the frame criteria and could not be made sense of in terms of the pre-existing issue frames.
