# Supplementary material for: Biochar in the UK Print News Media: Issue Frames and Their Implications for Opening up Debate About Land-based Greenhouse Gas Removal
Source: Environ Commun. 2024 May 29;18(8):1168–85. doi: 10.1080/17524032.2024.2357318 (PMC11574815; doi:10.1080/17524032.2024.2357318)
Supplement: Supplementary Material [file RENC_A_2357318_SM4414.pdf]

Supplementary material of Morris et al., "Biochar in the UK print news media: Issue frames and their implications for opening up debate about land-based greenhouse gas removal", *Environmental Communication*, 2024

Newspaper articles cited in the manuscript

Ahmed, N., 2014. Leaked IPCC climate plan to worsen global warming - ecologists. Guardian 7.4.2014.

Asprem, M., 2009. Most efficient way to produce biochar. Financial Times 7.3.2009.

Berglof, A., 2011. The long-lasting, eco-friendly; Could biochar be a revolutionary compost additive? Financial Times. 21.5.2011.

Connor, S., 2015. Climate change. Independent 6.12.2015.

Cookson, C., 2009. Green sky thinking to unlock terrestrial secrets. Financial Times 19.1.2009.

Cox, L., 2021. Environmentalists vow to block woodchip export plan in NSW Hunter region Guardian 8.9.2021.

Crooks, E., 2009. Five fighters of climate change. Financial Times 19.3.2009.

Diacono, M., 2011. We need to talk about peat. Telegraph 11.6.2011.

Dudman, J., 2010. UK government urged to evaluate biochar potential with trial schemes. Guardian 12.8.2010.

Flannery, T., 2015a. Climate crisis: seaweed, coffee and cement could save the planet. Guardian 20.11.2015.

Flannery, T., 2015b. Cop 21. Independent 12.12.2015.

Goodall, C., 2008. Green living: The 10 big energy myths. The Guardian 27.11.2008.

Guardian, 2009a. 'Biochar' goes industrial with giant microwaves to lock carbon in charcoal. Guardian 13.3.2009.

Guardian, 2009b. Extraordinary climate solutions presented in Manchester. Guardian 5.7.2009.

Guardian, 2009c. Turning charcoal into Carbon Gold. Guardian 27.8.2009.

Harries, R., 2009. His dark materials. Independent 27.9.2009.

Harvey, F., 2009. Can You Dig It? Financial Times 28.2.2009.

Harvey, F., 2019. Farming could be absorber of carbon by 2050, says report. Guardian 21.10.2019.

Leendertz, L. 2013. A slow burn success. Telegraph 6.4.2013.

Monbiot, G., 2009a. Woodchips with everything. Guardian 24.3.2009.

Monbiot, G., 2009b. Charleaders must cool enthusiasm for setting fire to the planet Guardian. 27.3.2009.

Murray, J., 2019. This dark material. Guardian 29.11.2019.

Nex, S., 2021. How to sort the snake oil from the sustainable. Telegraph 26.2.2021.

Nogrady, B., 2017. Negative emissions tech. Guardian 5.5.2017.

Preston, A., 2019. Wild at heart. Telegraph 29.6.2019.

Read, P., 2009. This gift of nature is the best way to save us from climate catastrophe. Guardian 27.3.2009.

Rughani, D., 2009. Reply – credit and criticism for biochar. Guardian 28.3.2009.

Sams, C., 2015. Carbon sinks will tip the market balance in favour of sustainable agriculture Financial Times 18.12.2015.

Sams, C., 2018. Carbon pricing can fix the broken food supply. Financial Times 19.9.2018.

Shemkus, S., 2015. Drive your car, help fight climate change? The Guardian 29.5.2015.

Telegraph, 2016. ‘Smoke and mirrors’ gardening? Telegraph 19.3.2016.

Thompson, K., 2016. Biochar: it’s not all black and white. Telegraph 11.6.2016.

Thornhill, J., 2020. Venture capital investors seek openings in climate tech to save the planet, and make money too. Financial Times 5.10.2020.

Thornhill, J., 2021. Climate tech 2.0 must sell venture capital on its future. Financial Times 22.10.2021

Vernon, J., 2010. Healthy soil a plus for everyone. Daily Telegraph 16.10.2010.

Vernon, J., 2011. The great autumn tidy up. Telegraph 1.10.2011.

Walker, J., 2012. For peat’s sake. Guardian. 16.6.2012.
